# The Daily Movement Pattern and Fulfilment of Physical Activity Recommendations in Swedish Middle-Aged Adults: The SCAPIS Pilot Study

**DOI:** 10.1371/journal.pone.0126336

**Published:** 2015-05-13

**Authors:** Elin Ekblom-Bak, Gustav Olsson, Örjan Ekblom, Björn Ekblom, Göran Bergström, Mats Börjesson

**Affiliations:** 1 Åstrand Laboratory of Work Physiology, The Swedish School of Sport and Health Sciences, Stockholm, Sweden; 2 Sahlgrenska Centre for Cardiovascular and Metabolic Research, Wallenberg Laboratory, Sahlgrenska University Hospital, Gothenburg, Sweden; 3 Department of Molecular and Clinical Medicine, University of Gothenburg, Gothenburg, Sweden; 4 Department of Cardiology, Karolinska University Hospital, Stockholm, Sweden; Institute of Preventive Medicine, DENMARK

## Abstract

Different aspects of the daily movement pattern—sitting, light intensity physical activity, and moderate- and vigorous intensity physical activity—have each independently been associated with health and longevity. Previous knowledge of the amount and distribution of these aspects in the general Swedish population, as well as the fulfilment rate of physical activity recommendations, mainly relies on self-reported data. More detailed data assessed with objective methods is needed. The aim of the study was to present descriptive data on the daily movement pattern in a middle-aged Swedish population assessed by hip-worn accelerometers. The cohort consisted of 948 participants (51% women), aged 50 to 64 years, from the Swedish CArdioPulmonary bioImage pilot Study. In the total sample, 60.5% of accelerometer wear time was spent sitting, 35.2% in light physical activity and 3.9% in moderate- and vigorous physical activity. Men and participants with high educational level spent a larger proportion of time sitting, compared to women and participants with low educational level. Men and participants with a high educational level spent more time, and the oldest age-group spent less time, in moderate- and vigorous physical activity. Only 7.1% of the study population met the current national physical activity recommendations, with no gender, age or education level differences. Assessment of all three components of the daily movement pattern is of high clinical relevance and should be included in future research. As the fulfilment of national physical activity recommendations is very low and sitting time is very high in our middle-aged population, the great challenge remains to enhance the implementation of methods to increase the level of physical activity in this population.

## Introduction

Physical activity (PA) is inversely associated with cardiovascular morbidity, as well as with total and cardiovascular mortality [1]. This may partly be mediated via positive effects of regular PA on several cardiovascular risk factors [2]. Hence, regular PA is important in modern health care, both for prevention and treatment.

Recently, excessive sitting has been recognized to be associated with metabolic risk, cardiovascular morbidity and mortality, independently of regular exercise [3, 4]. A sedentary behaviour is mainly limiting the light intensity PA (LIPA) embedded into daily life, but is poorly correlated with time spent in moderate- and vigorous intensity PA (MVPA) [5]. Therefore, it is highly relevant to evaluate all different aspects of the daily movement pattern (sitting, LIPA and MVPA).

National and international recommended PA levels are uniform worldwide, typically at least 150 minutes per week of MVPA, in bouts of 10 minutes or more, on preferable most days of the weeks [6, 7]. Some guidelines, for example in Sweden and Australia, have also incorporated recommendations on daily sitting [7, 8]. Previous studies have mainly relied on self-reported data, typically finding relatively high fulfilment of PA recommendations, but the increasing use of more objective movement monitors (e.g. accelerometers) has indicated a much lower fulfilment of PA recommendations [9, 10]. This is partly due to more strict analysis criterion relying on bouts and minutes derived from the objective captured data.

Using an objective PA assessment method provides the opportunity to capture different aspects of the daily movement pattern, which includes the volume and distribution of both activity and sedentary time throughout the day. Existing studies assessing the daily movement pattern by accelerometer recordings have shown similar overall figures for total time spent in sedentary, LIPA and MVPA, respectively, in different countries including the USA, Australia, Norway and Sweden [11–13]. In Sweden, a population based study on 18 to 75 year-old women and men has broadly studied the PA pattern, reporting a high proportion of daily time spent sedentary and a low fulfilment rate of national PA recommendations [9, 14]. However, more detailed data is lacking regarding the amount and distribution of the different aspects of the daily movement pattern in the Swedish middle-aged population. This is especially important as this target population is the most burdened by major lifestyle-related diseases (i.e. cardiovascular diseases). In addition, the proportion of the middle-aged population currently reaching national recommended levels, assessed by objective accelerometer data, is unknown.

The aim of the present study was therefore to present descriptive data on middle-aged, urban Swedish men and women aged 50 to 64 years, including the amount and distribution of PA and sedentary behaviour pattern, assessed by accelerometer. This also includes analysis of fulfilment of national recommendations in this population. A second aim was to analyse potential subgroup variations, with regard to gender, age and educational level.

## Materials and Methods

### Study population

The Swedish CArdioPulmonary bioImage Study (SCAPIS) is a major national effort to create a unique Swedish cohort for studies on cardiovascular disease, chronic obstructive pulmonary disease and related metabolic disorders [15]. In 2012, a comprehensive pilot study was conducted at the Sahlgrenska University Hospital in Göteborg, Sweden. A population sample including 2243 adults aged 50 to 64 years were randomly selected from the local peoples register, stratified for low and high socioeconomic status areas in the city of Gothenburg. Out of these, 1111 (50% women) agreed to participate. During two days, the participants underwent extensive imaging and functional studies of the heart, lungs and metabolism. They also filled in an extensive questionnaire including life style and living conditions and performed a submaximal cycle test to estimate cardiorespiratory fitness. The participants were asked to wear an accelerometer during seven days to objectively register the daily movement pattern. The study was approved by the ethics board at Umeå University (“Regionala etikprövningsnämnden Umeå”, Dnr 2010-228-31M) and it adheres to the Declaration of Helsinki. All participants provided written informed consent.

### Measurement of physical activity and sedentary behaviour

ActiGraph accelerometers (model GT3X and GT3X+, ActiGraph LCC, Pensacola, FL, USA) were used to objectively measure the daily movement pattern. A strong agreement between the two accelerometer models has previously been reported [16], enabling them to be used interchangeably within the same study. The ActiGraph accelerometer is a small (3.8 x 3.7 x 1.8 cm), lightweight (27 g) electronic device which records the acceleration of the participant´s movement. Subsequently, it provides an objective record of the intensity, frequency and duration of PA as well as sedentary behaviour, summarized in units called counts.

The accelerometer was handed to the participants during the first day of visits to the test center. The participants were instructed to wear the accelerometer in an elastic belt over the right hip during all waking hours for at least 7 consecutive days, except during water based activities. After the registration period, it was returned to the laboratory in a prepaid envelope. The accelerometer was initialized, data were downloaded and processed using the ActiLife v.6.10.1 software. Raw data sampling frequency was set to 30 Hz, and extracted as 60-s epoch with low frequency extension filter for the present analyses. Uniaxial (vertical axis) analyses were performed to permit comparisons with previous presented research.

#### Data processing

A total of 1067 participants (96.0%), agreed to wear an accelerometer. Minimum requirement for data inclusion was 600 minutes of valid daily monitor wear on at least 4 days [17]. Wear time was defined by subtracting non-wear time from 24 hours. Non-wear time was defined as at least 60 consecutive minutes with no movement (0 cpm), with allowance for maximum 2 minutes of counts between 0 and 100 [18]. In total, 7 accelerometers were lost by participants or in mail transport and another 112 provided invalid data due to above wear time restriction (n = 68), labelling error (n = 41), or accelerometer malfunction (n = 3). The majority, 65%, of the included participants had valid data for at least 7 days, and a further 21% for 6 days, 9% for 5 days, and 5% for 4 days. Thus, 948 individuals or 85.3% of the randomly selected population showed valid data, and were included in the present analyses.

As wear time varied between study participants above the minimum of 600 minutes per day, the daily movement pattern is presented as percentage of wear time spent in different intensity-specific categories. Absence of, or very low registrations (<100 cpm) was recognized as being sedentary [19], while cpm between 100 and 2019 were defined as LIPA [18], and cpm ≥2020 as MVPA [18] (with no further distinction between moderate and vigorous PA). Moreover, mean counts per minute (cpm) and time spent in MVPA is presented. Mean cpm is a measure of overall daily activity intensity, and expressed as the total numbers of counts divided by minutes of wear time. Time in MVPA was analysed as total accumulated minutes of MVPA, or as MVPA minutes accumulated in sustained bouts of ≥10 consecutive minutes above threshold, with allowance of interruption up to 2 minutes below threshold.

Specific analyses of sedentary behaviour characteristics were carried out, including the number of and total time in sedentary bouts as well as breaks per sedentary hour. Expressing breaks from sedentary as the rate per sedentary hour, is suggested to be a more relevant metric for free-living behaviour compared to absolute number of breaks [20]. A prolonged sedentary bout was defined as ≥20 minutes of cpm below 100, with no allowance for interruption above threshold (a definition which previously have been reported to associate with clinical changes in cardio-metabolic biomarkers, blood pressure and gene expression [21–23]). A sedentary break was defined as an interruption in sedentary time from one minute of <100 cpm, to the following minute ≥ 100 cpm [24].

The percentage of the study population meeting different defined MVPA recommendations was also analysed. Current Swedish national guidelines recommend at least 150 minutes per week of MVPA in bouts of 10 minutes or more preferably on most days of the weeks (5 out of 7) [7]. Hence, these recommendations require regular accumulation of MVPA during the week as well as MVPA minutes accumulated in prolonged bouts. To catch this complexity, we evaluated the percentage of the study population meeting the criterion of 150 minutes of MVPA per week with a variation in the requirement of regularity and/or accumulation in prolonged bouts, according to the following criteria; 1) accumulating 150 minutes per week, 2) accumulating 150 minutes per week, of which all are from prolonged bouts of 10 minutes or more, 3) accumulating 30 minutes per day on at least 5 of 7 days of the week, and 4) accumulating 30 minutes per day on at least 5 of 7 days of the week, of which all are from prolonged bouts of 10 minutes or more. For participants with less than 7 days of valid data, the requirement for fulfilment was at least 4 out of 6 days, or 4 out of 5 days, or 3 out of 4 days. Importantly, the last two categories are reflecting the current national PA recommendations, depending on the different possible interpretations of these, i.e. “preferably most days of the week”, requiring 5–7 days of activity or not.

### Other measurements

Measurements of weight, height and waist circumference were assessed during the first visit to the test centre. Body mass index (BMI) was computed as weight (kilograms) divided by square height (meter^2^). Age was categorized into 3 levels (50 to 54, 55 to 59, and 60 to 64 years). Through self-administrated questionnaire responses, education level was dichotomized into high or low (university degree or not), and smoking habits into being a regular smoker or not. The SCAPIS pilot study used the same questions to assess self-reported sedentary leisure time and PA habits as the Public Health Agency of Sweden did to screen the entire population. Using the same questions enabled comparison of the self-reported daily movement pattern in the SCAPIS pilot study population with Swedish national data. For assessment of sedentary leisure time, the responses to a question regarding leisure time activity habits the past 12 months were dichotomized into highly sedentary leisure time (option 1, “sedentary leisure time”) or not (option 2, 3 and 4, representing exercise for two hours a week without sweating up to regular exercise and training at least 3 times a week). Questions assessing exercise habits were used to dichotomize participants into accumulating at least 30 minutes of daily PA or not.

### Statistical analysis

Data was checked for normality using the Shapiro-Wilk test. The majority of the variables were skewed, and hence the descriptive data is presented as proportions or median and 25^th^-75^th^ percentile (Q1-Q3). A 2-sample z-test was used to compare proportional differences between the study population and the Swedish national data, as well as potentional differences between gender, age-groups, and educational level for fulfilment of the different MVPA recommendations. General linear modeling was used to pairwise compare the estimated marginal means (means adjusted for by the covariates in the model) for daily percentage of time spent in sedentary, in LIPA and MVPA, mean cpm, total wear time, and the variables in the sedentary pattern analysis between men and women, three age-groups (50–54, 55–59 and 60–65 years) and low and high education level, respectively. Interactions were identified when the 95% CI for the mean difference was not including zero. All skewed variables were transformed to fit normal distribution before introduced into the general linear model analysis. In the descriptive text and tables, non-transformed data is presented. The level of significance was set at p<0.05 for all analyses. Adjustment for multiple testing between age-groups was made by using significance level of p<0.0025 for proportional comparison, and Bonferroni confidence interval adjustment in the general linear modeling. A related-samples ANOVA for nonparametric data was used to test for variation of daily percentage of time spent in sedentary, LIPA and MVPA over the week, and an independent Mann-Whitney U test to compare daily percentage of time spent in sedentary, LIPA and MVPA for each day of the week between genders. All statistical analyses were performed using SPSS (Statistical Package for the Social Sciences for Windows, 14.0, 2006, SPSS Inc., Chicago IL).

## Results

A total of 948 participants (51% women) were included in the analyses. Overall, the median (Q1-Q3) age was 57.5 (53.7–61.7) years and 69% of the participants were classified as being overweight or obese (Table 1). Thirty-eight percent had university degree and 12% were smokers on regular basis. Compared with Swedish national data in the age-group 45 to 64 years from year 2012, a higher proportion of men and women in the present study were classified as overweight or obese (BMI > 25, Note: weight and height were measured in the present study, but self-reported in the Swedish population data). Women in the present study smoked less. Otherwise, there were no significant differences regarding education level, smoking habits in men, the proportion reporting a highly sedentary leisure time and accumulating at least 30 minutes of daily PA, respectively.

**Table 1:** Characteristics of the study population and Swedish population data with regard to gender.

	Study population		Swedish population data*	
	Men (n = 462)	Women (n = 486)	Men	Women
Age (years)	57.7 (53.8–62.0)	57.5 (53.7–61.4)		
Weight (kg)	86.6 (79.5–95.0)	70.4 (63.7–80.0)^a^		
Height (cm)	178 (173–183)	165 (160–169)^a^		
Waist (cm)	99 (94–105)	89 (81–98)^a^		
BMI (kg/m^2^)	27.1 (25.1–29.3)	26 (23.4–29)^a^		
High education level (% university degree)	35	41^a^	32	38
BMI^#^ ≥25 (%)	77	60^a^	65^b^	47^b^
Regular/Daily smoker (%)	13	11	15	19^b^
Highly sedentary during leisure (%)	16	15	13	13
30 min of daily physical activity, self-reported (%)	65	61	64	63

Data presented as median (Q1-Q3)

BMI = Body Mass Index

* Swedish population data for the age group 45–64 years for year 2012. Data for education level, BMI and smoking are from Statistics Sweden (freely available at www.scb.se). Data for the self-reported sedentary and physical activity behaviour are from the Public Health Agency of Sweden (freely available at www.folkhalsomyndigheten.se).

^#^ Weight and height were measured in the present study population, but self-reported in the Swedish population data.

^a^ Significant gender difference (p<0.05, independent Mann-Whitney U test).

^b^ Significant proportion difference between the present study population and Swedish population data, p<0.05.

Daily median wear time was 14.3 h per day, with no significant differences between the subgroups included in the analyses. The study participants (of both genders) spent 60.5% of the wear time sitting, with men and participants with high educational level spending a larger proportion of time sitting, compared to women and individuals defined as having a low educational level (Table 2). The proportion of time being sedentary, correlated negatively with LIPA (spearman rho = -0.96), and hence similar significant associations as for sedentary, but opposite, was found for LIPA. Moreover, men and participants with a high educational level, spent more time, and the oldest age-group spent less time, in MVPA. There were no significant differences in overall PA (as mean cpm) observed, between gender and education level, while the oldest age-group had lower overall PA than the two younger age-groups.

**Table 2:** Daily movement pattern (% of wear time in different intensity categories) and mean cpm among all study participants and in subgroups.

	Sedentary (%)	LIPA (%)	MVPA (%)	Mean cpm
All	60.5 (54.5–66.4)	35.2 (29.9–41.1)	3.9 (2.4–5.6)	335.6 (263.7–422.9)
Gender				
Women	58.4 (53.2–64.8)	37.2 (31.5–42.2)	3.6 (2.2–5.2)	336.8 (265.8–414.2)
Men	62.0 (56.2–67.8)^a^	33.5 (28.0–38.5)^a^	4.1 (2.5–5.8)^a^	333.0 (262.5–433.3)
Age (yrs)				
50–54	60.9 (54.2–65.9)	35.1 (30.6–41.3)	3.9 (2.5–5.7)	344.6 (279.8–426.9)
55–59	59.2 (53.5–66.3)	36.1 (29.8–41.8)	4.2 (2.8–6.0)	456.4 (278.3–438.4)
60–65	61.4 (56.0–67.0)	34.6 (29.4–39.8)	3.3 (1.8–5.2)^b^ ^,^ ^c^	311.8 (224.5–398.5)^b^ ^,^ ^c^
Education level				
Low	59.8 (53.4–66.2)	36.3 (30.5–42.1)	3.7 (2.1–5.6)	330.9 (259.1–423.7)
High	61.3 (55.8–66.5)^d^	34.1 (29.2–38.9)^d^	4.1 (2.8–5.6)^d^	339.2 (277.8–421.2)

Values are median (Q1-Q3) of individual means.

cpm = counts per minute

^a^ Significant gender difference, p<0.05.

^b^ Significant age group difference vs 50–54 years, p<0.05.

^c^ Significant age group difference vs 55–59 years, p<0.05.

^d^ Significant EL difference, p<0.05.

All analyses comparing subgroups are adjusted for (when not evaluated) gender, age,

socioeconomic area, education level (EL) and wear time.

**Fig 1:**
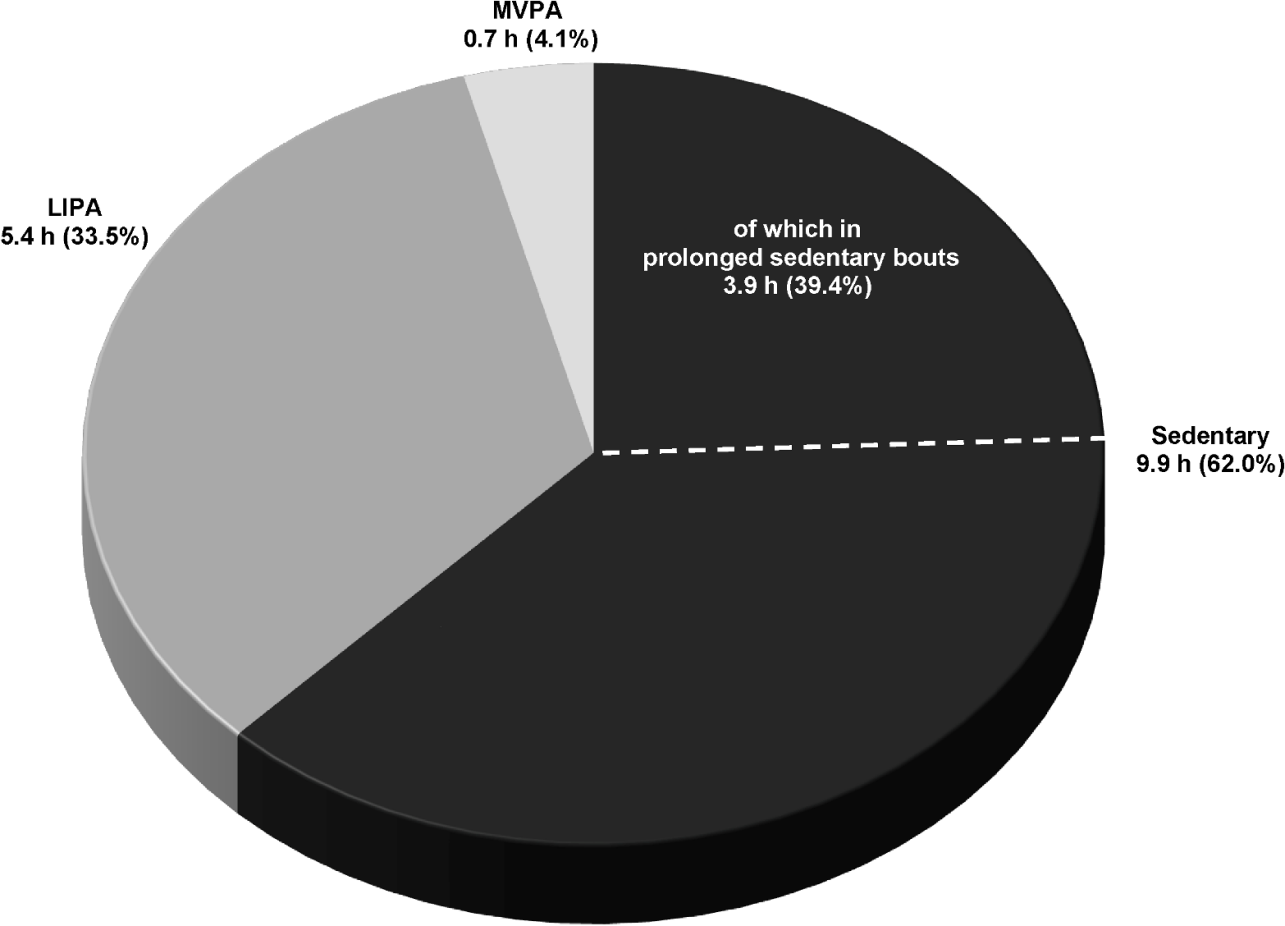
The distribution of the daily movement pattern in men of the study population. Time spent in sedentary (total and in prolonged bouts ≥ 20 minutes), low-intensity physical activity (LIPA) and moderate- and vigorous physical activity (MVPA) over hypothetically 16 awake hours in men of the study population. The calculation of hours is based on the objectively captured proportion of wear time spent in the different categories of the daily movement pattern.

**Fig 2:**
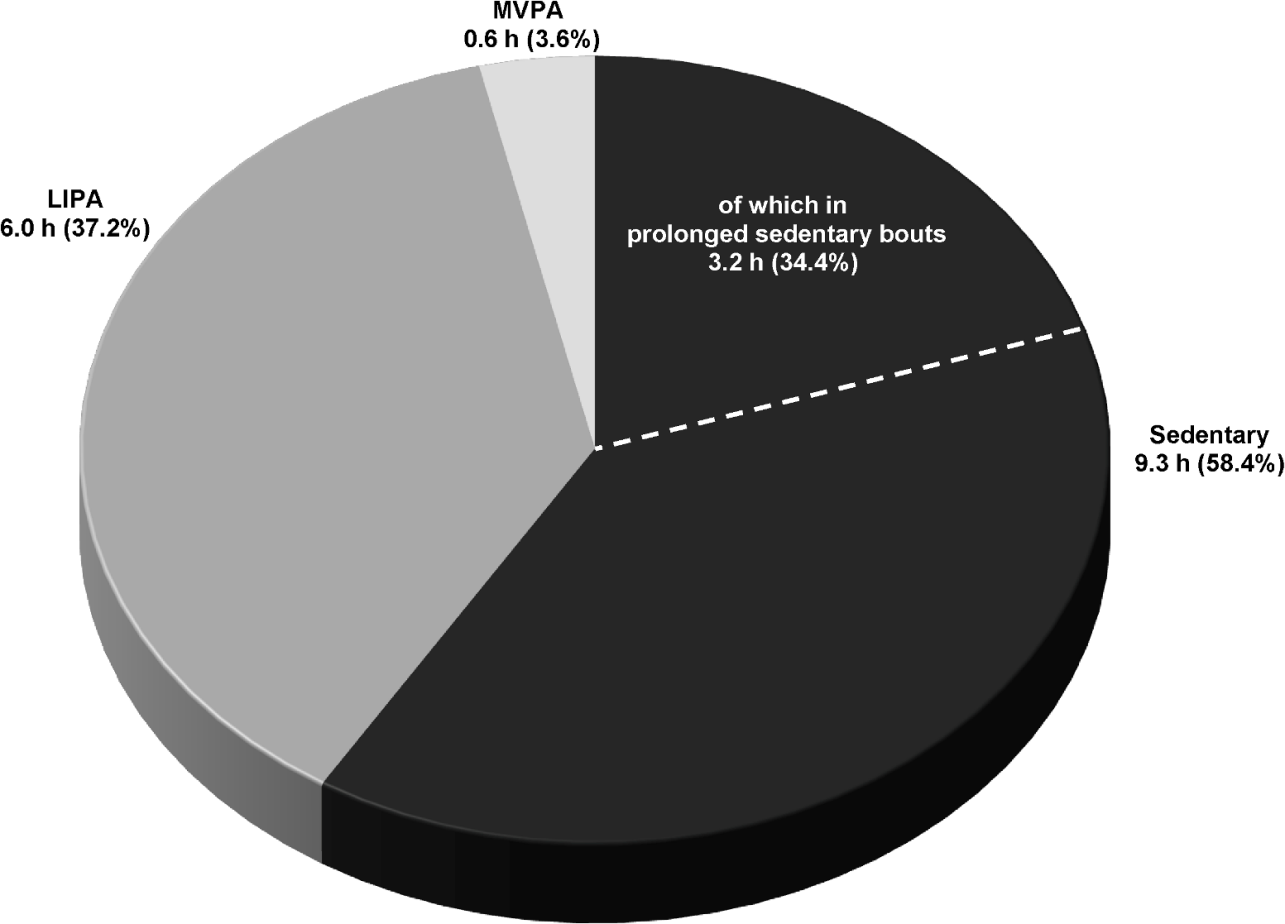
The distribution of the daily movement pattern in women of the study population. Time spent in sedentary (total and in prolonged bouts ≥ 20 minutes), low-intensity physical activity (LIPA) and moderate- and vigorous physical activity (MVPA) over hypothetically 16 awake hours in women of the study population. The calculation of hours is based on the objectively captured proportion of wear time spent in the different categories of the daily movement pattern.

In order to further illustrate the distribution of the daily movement pattern for men and women in the present study, Figs 1 and 2 presents the proportions of wear time spent in sedentary, LIPA and MVPA translated into daily hours from hypothetically 16 waking hours (assuming 8 hours of sleep) for men and women, respectively.

The above daily movement patterns are based on weekly average data. In Table 3, the percentages of wear time spent in sedentary, LIPA and MVPA specified by the days of the week for women and men, respectively, are presented. In line with the weekly average data, men spend significantly more time in sedentary and less time in LIPA on all days, compared to women. A strong trend of variation in sedentary and LIPA over the week was seen for both women and men (p<0.001). This was mainly due to variations between weekdays (Mon-Fri) and weekend days (Sat-Sun); for women 59.3% vs. 57.0% for sitting (p<0.001) and 36.7% vs. 38.5% for LIPA (p<0.001) and for men 63.1% vs. 60.5% for sitting (p<0.001) and 32.4% vs. 34.9% for LIPA (p<0.001). Regarding MVPA, there was a significant variation over the week for women, however, not due to variations between weekdays and the weekend (p = 0.441). When comparing the variation between weekdays and the weekend in participants with a high educational level, similar significant patterns were seen; 62.6% vs. 57.4% for sitting (p<0.001), 33.0% vs. 37.5% for LIPA (p<0.001), and 3.8% vs. 4.1% for MVPA (p = 0.025). For participants with a low educational level, only the proportion of time in MVPA differed between weekdays and weekends, 3.8% vs. 2.9% (0.032).

**Table 3:** Daily movement pattern (% of wear time in different intensity categories) presented per day of the week for women and men, respectively.

	Women				Men			
	n	Sedentary (%)	LIPA (%)	MVPA (%)	n	Sedentary (%)	LIPA (%)	MVPA (%)
Monday	452	60.8 (52.6–68.2)	35.3 (28.3–43.1)	2.9 (1.5–5.5)	420	64.6 (56.1–70.4)^a^	31.3 (25.3–38.4)^a^	3.8 (1.8–6.1)^a^
Tuesday	457	60.0 (52.0–68.1)	35.9 (28.6–43.9)	3.2 (1.6–5.6)	410	63.1 (53.7–70.7)^a^	31.8 (24.6–41.4)^a^	3.5 (1.9–6.4)
Wednesday	453	59.5 (51.9–67.5)	36.3 (28.5–43.0)	3.8 (1.9–6.1)	423	63.1 (54.8–70.6)^a^	32.2 (24.9–40.3)^a^	3.7 (2.0–6.3)
Thursday	451	59.3 (50.9–66.7)	37.1 (29.6–44.9)	3.1 (1.6–5.3)	430	63.7 (54.9–72.0)^a^	32.4 (24.8–39.9)^a^	3.4 (1.9–6.5)^a^
Friday	455	59.0 (51.8–66.8)	37.2 (29.7–44.2)	3.0 (1.6–5.2)	435	63.1 (55.5–71.1)^a^	32.1 (25.2–39.8)^a^	3.3 (1.7–5.7)
Saturday	440	57.1 (49.3–63.4)	38.7 (32.1–46.4)	2.8 (1.2–5.5)	384	61.2 (52.4–68.4)^a^	34.0 (27.8–42.0)^a^	3.4 (1.4–6.7)^a^
Sunday	419	57.7 (48.3–65.6)	38.1 (31.1–45.8)	2.7 (1.1–5.8)	369	60.4 (51.2–68.2)^a^	34.4 (27.6–43.0)^a^	3.3 (1.2–7.1)
p trend		<0.001	<0.001	0.023		<0.001	<0.001	0.537

Values are median (Q1-Q3) of individual means.

^a^ Significant gender difference, p<0.05.

Table 4 presents the sedentary behaviour characteristics. Overall, the entire study population had on average 5.8 prolonged bouts (≥20 minutes) of sitting per day, and spent 189 minutes (3.1 hours) in these prolonged bouts. Moreover, sitting time was broken up on average 10.1 times per sedentary hour. Compared to women, men had a greater number of prolonged sedentary bouts, spent 40 minutes more in prolonged bouts, and had less breaks per sedentary hour. Similar trends were seen in highly educated participants compared to participants with lower education. No significant differences were observed between age-groups (p>0.126).

**Table 4:** Characteristics of sedentary behaviour among all study participants and in subgroups.

	Daily average numbers of sedentary bouts^*^	Daily average time in sedentary bouts^*^ (min)	Numbers of breaks^#^ per sedentary hour
All	5.8 (4.3–7.6)	188.8 (136.3–252.6)	10.1 (8.5–11.8)
Gender			
Women	5.3 (4.1–7.1)	170.1 (127.3–235.2)	10.8 (9.1–12.3)
Men	6.4 (4.7–8.0)^a^	209.8 (146.2–267.8)^a^	9.5 (8.1–11.3)^a^
Age (y)			
50–54	5.8 (4.3–7.3)	189.0 (131.5–242.2)	10.2 (8.8–11.7)
55–59	5.7 (4.4–7.6)	183.4 (136.7–254.4)	10.1 (8.5–12.2)
60–65	6.0 (4.4–7.7)	194.0 (139.4–264.2)	9.9 (8.3–11.7)
Education level			
Low	5.6 (4.2–7.5)	179.2 (127.7–246.8)	10.4 (8.7–12.2)
High	6.1 (4.7–7.6)^e^	204.1 (150.1–259.1)^e^	9.6 (8.3–11.5)^e^

Values are median (Q1-Q3) of individual means.

* A sedentary bout is defined as ≥ 20 minutes of <100 cpm, with no allowance for interruption above threshold.

^#^ A sedentary break is considered interruption in sedentary time (minimum 1 min).

^a^ Significant gender difference, p<0.05.

^e^ Significant EL difference, p<0.05.

All analyses comparing subgroups are adjusted for (when not evaluated) gender, age, socioeconomic area, education level (EL) and wear time.

The median time of daily MVPA in the entire study population was 32.8 minutes, however only 9.5 minutes of those were accumulated in prolonged bouts (≥10 minutes) (Table 5). Women spent less total time in MVPA than men, while no gender difference regarding MVPA minutes accumulated in bouts, could be seen. Older participants spent less, and those highly educated more time, in total as well as in prolonged bouts of MVPA.

**Table 5:** Time in MVPA and fulfillment of different defined MVPA recommendations among all study participants and in subgroups.

			Fulfillment of different defined MVPA recommendations, accumulating at least			
	Daily average MVPA (min)	Daily average MVPA in bouts ≥10 min (min)	150 min/week	150 min/week in bouts of ≥10 min	30 min/day on most days of the week*	30 min/day in bouts of ≥10 min on most days of the week*
All	32.8 (19.9–48.3)	9.5 (2.6–21.4)	72.5%	25.1%	35.3%	7.1%
Gender						
Women	30.9 (19.3–45.6)	9.4 (2.6–20.4)	70.0%	23.7%	30.7%	8.0%
Men	35.4 (21.4–49.5)^a^	9.6 (2.7–22.1)	75.1%^a^	26.6%	40.3%^a^	6.0%
Age (y)						
50–54	32.8 (21.4–48.4)	9.3 (2.6–20.7)	75.7%	24.6%	34.1%	5.2%
55–59	36.0 (23.6–51.1)	11.4 (3.4–24.0)	78.3%	29.2%	41.8%^b^	8.1%
60–65	28.3 (16.1–45.5)^b,^ ^c^	8.2 (1.9–18.0)^c^	63.7%^b,^ ^c^	21.5%^c^	30.2%^c^	7.4%
Education level						
Low	31.1 (18.6–48.0)	8.0 (1.6–19.3)	67.5%	22.4%	34.6%	6.7%
High	35.3 (23.9–49.0)^d^	12.8 (5.6–23.4)^d^	80.8%^d^	29.7%^d^	36.7%	7.8%

Values are median (Q1-Q3) of individual means.^a^ Significant gender difference, p<0.05.

^c^ Significant age group difference vs 55–59 years, p<0.05.

^d^ Significant EL difference, p<0.05.

* 5 out of 7 days for participants with 7 valid days. For participants with less than 7 valid days, the requirement of fulfilment was at least 4 out of 6 days, or 4 out of 5 days, or 3 out of 4 days.

Analyses comparing subgroups for daily average MVPA and daily average MVPA in bouts ≥10 min are adjusted for (when not evaluated) gender, age, socioeconomic area, education level (EL) and wear time.

In total, 72.5% of the study population accumulated at least 150 minutes per week of MVPA, when neither prolonged nor regular MVPA during the week were required (Table 5). With requirement of MVPA being accumulated either in prolonged bouts or regularly throughout the week (on at least 5 of 7 days), the proportion dropped to 25.1% and 35.3%, respectively. When both prolonged and regular MVPA throughout the week were required, in concordance with the “toughest” interpretation of current national guidelines (30 minutes per day in bouts of ≥ 10 minutes on at least 5 of 7 days of the week), only 7.1% of the study population met the criteria. In subgroups, a larger proportion of men compared to women met the criteria when no requirement of prolonged bouts of MVPA was set. Participants in the oldest age-group fulfiled the different recommendations to a lower extent. This was also seen in those with low educational level, for the least demanding recommendation. However, for the recommendations in concordance with the current national guidelines, there were no subgroup differences.

## Discussion

The aim of this paper was to describe the daily movement pattern, including the sedentary characteristics, and fulfilment of the national PA recommendations, in an urban Swedish middle-aged population with regard to gender, age and educational level. A main finding was that almost two-thirds of the accelerometer wear time was spent sedentary, with more than a third of sedentary time being spent in prolonged bouts. This means that in relation to the study population´s average wear time (14.3 hours), on average 8 hours and 40 min was spent sedentary, of which more than 3 hours were in prolonged bouts. The other main finding was that we found a low fulfilment of the national PA recommendations, with only 7.1% of the population meeting the current Swedish recommendations of weekly MVPA. With a good agreement between key variables in the present unselected study population and Swedish national population data, these results may reflect the activity and sedentary pattern of women and men of similar age in the Swedish population.

### Daily movement pattern

Previous research describing the daily movement pattern by accelerometer recordings in representative population samples has been sparse. Together with variations in wear time definition, cut-offs used to identify intensity-specific categories and selection of age-groups in the presentation of the data in previously published data, complicate comparisons with the present results.

We found that 60.5% of accelerometer wear time was spent sedentary, 35.2% in LIPA and 3.9% in MVPA, with an overall activity of 335.6 cpm. Regarding MVPA, this percentage equals to approximately 33 minutes of total MVPA per day. However, only 9.5 of those minutes were accumulated in prolonged bouts of MVPA (<10 min). These figures are in concordance with earlier data in Swedish men and women of similar age-groups (40 to 59 and 60 to 75 yrs) collected in 2008 [14]. The same authors also reported an average increase, in the proportion of time spent in sedentary, since 2002, by 4.2% and 6.9% for the two age-groups, respectively. This corresponds to an increase of 18 and 36 minutes in daily sedentary time, to 516 and 532 minutes per day in 2008, for respective group. However, in a small sample of Australian men and women, aged 30 to 87 years [12], less time than in the present study was spent being sedentary (57%) and more time in LIPA (39%), with a similar proportion being spent in MVPA (4%). Data from the 2003–2004 NHANES study on a representative sample of US adults indicated that they spent slightly less time in sedentary (58.9%) and more time in LIPA (39.0%), than found in the present study. That said, they also spent less time in total MVPA (2.2%) [11]. In a population based study in Norwegian 50 to 64 year-old men and women, 62.3% of daily wear time was spent sedentary [25]. No further comparisons between the studies, regarding wear time spent in LIPA and MVPA are feasible, as different cut-offs defining those intensity categories were used.

Gender differences in the daily movement patterns were observed. Men spent more time sedentary, with subsequently less time in LIPA, however, with more time in total MVPA. This resulted in no significant gender difference regarding total daily activity, assessed as mean cpm. Similar gender differences have previously been reported in several studies [11, 13, 14, 18, 25].

The trends of higher sedentary time and lower time spent in MVPA in the oldest age-group (60–64 yrs) compared to the youngest (50–54 yrs), resulting in lower mean cpm for the older participants, are also in line with previous reports [11, 26]. Moreover, participants with a higher educational level spent significantly more time being sedentary, but also a higher time in both total and prolonged bouts of MVPA, a pattern previously described [25, 27]. There were some variations in movement pattern comparing weekdays and weekends. On weekends, participants with a high educational level spent significantly less time sedentary, and more time in LIPA and MVPA than on weekdays. This may suggest that participants with a higher education are more likely to have an occupational setting during the weekdays, requiring a greater volume of sedentary (office-based occupations, computers or other labour saving devices etc.), while being more regular physical active days out of office (so called “weekend warriors”). This is in line with results from Australian office workers, in which a higher sitting time, less LIPA and more time in MVPA was seen when comparing a work and a non-work day, as well as work hours and non-work hours on work day [28]. Notably, participants with low educational level spent less time in MVPA on the weekend compared to weekdays. In the present analyses, however, we were not able to distinguish between work hours and non-work hours as no individual log to specify this was applied.

### Sedentary behaviour characteristics

Regular interruptions in sedentary time by short bouts of light-intensity activity have been associated with a more healthy risk profile, regarding waist circumference, triglyceride levels, 2-hour plasma glucose and C-reactive protein levels [3, 24], as well as postprandial glucose and insulin response, arterial function, blood pressure and gene expression [21, 22, 29]. Also, the accumulation of sedentary time in prolonged bouts (at least 10 minutes) has been shown to be more strongly related to metabolic risk profile, compared to sedentary time in shorter bouts [30]. In the present study, men did not only spend a large proportion of total wear time being sedentary, they also spent approximately 40 more minutes in prolonged sedentary bouts and had less breaks per sedentary hour than women. Similar patterns were seen in participants with a high educational level. These findings highlight men and individuals with higher education level (especially during office-hours) as especially important targets for future interventions regarding PA.

### Fulfilment of PA recommendations

The present results show a high variation in fulfilment of current PA recommendations. Only 7% of the study population of middle-aged Swedes met the criteria for national PA recommendations (at least 150 minutes per week of MVPA on preferable most days of the weeks, in bouts of 10 minutes or more). Although, very low, this proportion is slightly higher than previously reported in a mixed age-sample of Swedish adults, 1% [9], highlighting that as much as 93% of middle-age adults might not engage in recommended amounts of regular physical exercise. Importantly, these figures are entirely different from data typically obtained from surveys, using self-reported questionnaires. Interestingly, there were no subgroup differences (gender, age, educational level) in the proportion meeting the recommendations. Though, a simple evaluation of adherence to PA recommendations derived by objective accelerometer recordings is not completely correct, as mainly self-report questionnaire data was used to develop current recommendations. Along with the increased use of accelerometry in epidemiological research, there may be a need in the future for new recommendations based on objective data.

In the full study population as well as in the subgroup analysis, the previously phenomenon of an “active couch potato” was spotted [31]. This refers to those individuals who achieve sufficient amount of daily MVPA according to public health recommendations, but also spend most waking hours sedentary. Although the proportion of the wear time spent being sedentary was lower in the subgroup of participants meeting current MVPA recommendation, compared to the rest of the sample (56.5% vs. 60.9%), still the majority of wear time hours was spent sedentary also in these otherwise active participants.

The present results are interesting in the light of recent publications based on self-report, which report high levels of PA, a high degree of sporting activity and exercise, and little sedentary time [32, 33]. For example, the Swedish middle-aged population in the Eurobarometer [27] reported on average 6 hours spent being sedentary, while in the present study population, objectively assessed time spent sedentary was almost 9 hours. The low validity of questionnaires for PA assessment is well known, but sometimes neglected. Nevertheless, media do often spread the news of high exercise/sporting activity, while in fact, the overall activity level in the population remains rather low. Speculatively, this incongruence may at least partly reflect a higher social desirability to report being physically active, in recent years.

The high proportion of sedentary time and low levels of MVPA recommendation fulfilment in the present study population, are highly clinically relevant findings. This is not least because PA levels are might decrease further in the future. A recent study based on historical trend data from the UK, forecast an increase in weekly sedentary time by approximately 10 hours until the year 2030, with subsequent decrease in PA in all domains (leisure, travel, occupational and domestic), expressed as average MET-hours per week [34]. Implementation of methods to decrease the time spent sedentary and to increase PA-levels, is needed. One such efficacious method is the Swedish model of PA on prescription (PAP), which has been shown to increase the level of PA [35], quality-of-life, as well as have a positive effect on cardiometabolic markers [36]. Recently, the Swedish National Bureau of Health and Welfare produced in 2011 guidelines stating that the Health Care system should provide structural activity counselling to all insufficiently physically active patients. As the present study show, the number of insufficiently active individuals may be very large.

### Strength and limitations

The strengths of the present study include a representative sample of the randomly selected study population, a low dropout rate and high proportion of participants with valid accelerometer data. Moreover, the objectively assessed time spent in sedentary, LIPA and MVPA gives a more valid estimate of actual daily movement pattern than self-report methods do. The accelerometer data was collected throughout the year, limiting systematical bias of season variability in PA and sedentary habits. Limitations include the inability of the accelerometer to differentiate between sitting and standing as well as the automated wear time estimation used, as low counts during 60 minutes may be common in this age-group. However, the latter was chosen as no individual wear time logs were available, and to enable comparison with previous published data using the same wear time estimation. In addition, caution must be applied when extrapolating the presented data to men and women, outside the ages of the study participants. Also, although the comparisons with Swedish national data indicated small difference between the SCAPIS pilot cohort and men and women of corresponding age in the Swedish population, extrapolation of the results to other parts of Sweden should be done with caution.

## Conclusions

The main findings of the present study were that about two-thirds of the daily time in a large sample of Swedish middle-aged population, was spent being sedentary and that only a minority (7%) of the study participants met the current national PA recommendations.

The daily time being sedentary, as assessed by accelerometry, are several hours higher than previously self-reported sedentary time in questionnaires.

The present study highlights the importance of a comprehensive assessment of individual daily movement pattern. Importantly, the variations of the time spent being sedentary, LIPA and MVPA, were not reflected in the overall activity recorded in the present study (mean cpm). Therefore, assessment of all three components of the daily movement pattern is of high clinical relevance and should be included in future research.

As the fulfilment of national PA recommendations is very low and sedentary time is very high in our middle-aged population, the great challenge remains to enhance the implementation of methods to increase the level of PA in this population.
